# Defining the minimal peptide sequence of the ING1b tumour suppressor capable of efficiently inducing apoptosis

**DOI:** 10.1038/cddiscovery.2015.48

**Published:** 2015-10-26

**Authors:** A Boyko, K Riabowol

**Affiliations:** 1 Departments of Biochemistry and Molecular Biology, University of Calgary, Calgary, AB, Canada; 2 Department of Oncology, University of Calgary, Calgary, AB, Canada

## Abstract

The ING1b protein is a type-II tumour suppressor and stoichiometric member of the Sin3 histone deacetylase (HDAC) protein complex in which it acts to target HDAC activity to regulate chromatin structure. Altering ING1 levels by ectopic expression of ING1b in cancer cells promotes apoptosis, whereas altering levels by knockout in normal murine fibroblasts alters sensitivity to doxorubicin-induced apoptosis. We have identified a minimal region of ING1b capable of inducing levels of apoptosis in targeted cells as effectively as full-length ING1b, using transient overexpression of ING1b fragments followed by the Annexin V assay. We observed high levels of apoptosis in 14 of 14 cancer cell lines tested. Infecting triple-negative tumorigenic MDA-MB-468 breast cancer, U2OS or Saos-2 cells at multiplicities of infection (MOIs) ranging from 10 to 20 rapidly triggered apoptosis in ~80% of infected cells within 48 h. This was not due to the effects of virus, as infection at the same MOI with a control adenovirus expressing GFP was not effective in inducing apoptosis. When used at low MOIs, the ING1b fragment showed a cell-killing efficacy that was higher than native, full-length ING1b. Using a doxycycline-regulated inducible p53 expression system demonstrated that apoptosis induced by the ING1b fragment was p53 independent. Given the growing importance of combination therapies, we evaluated whether there was synergism between the ING1b fragment and HDAC inhibitors. Combination treatments with TSA, LBH 589 and SAHA reduced cancer cell survival by 3.9–4.7-fold as compared with single-drug treatment, and resulted in ~90% reduction in cell survival. Normalized isobologram analysis confirmed strong synergism between the ING1b fragment and drugs tested. These findings provide support for using ING1b-derived therapeutics as adjuvant treatments in combination with existing epigenetic therapies.

## Introduction

The inhibitor of growth (ING) family of type-II tumour suppressors is comprised of five genes encoding multiple isoforms. All INGs display a high degree of evolutionary and functional conservation and are present in species ranging from yeast to humans.^1,2^ They function as stoichiometric members of histone acetyltransferase (HAT) and histone deacetylase (HDAC) protein complexes and share a number of conserved protein domains that largely determine their molecular function as readers and writers of the histone code. In addition to, or as a consequence of their function as epigenetic regulators, they affect DNA repair, apoptosis, cellular senescence and proliferation. ING1b is the best-studied member of ING family and is predominantly found in Sin3A HDAC1- and HDAC2-containing complexes,^3^ where it mediates recruitment of these complexes to chromatin targets.^4^ Recently, ING1b was also shown to function in gene-specific DNA demethylation,^5^ and to regulate gene expression by modulating microRNA biogenesis.^6^ Consistent with their designation as tumour suppressors, a large number of clinical studies have reported complete or partial loss of ING1b expression in different type of tumours.^7–9^

Induction of apoptosis in human tumours by restoration or augmentation of pathways disrupted in cancer cells, is often considered a main objective when developing new cancer treatment strategies. The ability of ING1b to induce apoptosis is well documented.^10–14^ It relies on multiple molecular mechanisms and occurs in both p53-dependent and p53-independent manners.^12,15–18^ Upregulation of ING1b and p53 during apoptosis is associated with increased bax levels and altered mitochondrial membrane potential, suggesting that they may induce apoptosis, in part, via the intrinsic mitochondrial cell death pathway.^19^ Similarly, ING interactions with p53 and CSIG proteins^12,20^ lead to apoptotic signalling via an intrinsic apoptosis pathway; upregulation of bax gene expression and cytochrome C release followed by caspase activation. Furthermore, past studies have demonstrated that ING1b can sensitize cells to the extrinsic apoptosis pathway through induction of the heat shock protein HSP70 followed by TNF-*α*-mediated apoptosis.^11^ Indeed, a number of independent studies reported significant pro-apoptotic effects triggered by ectopic expression of ING1b in cell culture and animal tumour models using various delivery systems.^19,21–23^ A strong synergistic response resulting in reduced tumour volume was observed when ectopic ING1b expression was combined with 5-azacytidine treatment in a mouse xenograft model.^22^ In our study, we have used what is known about ING domains in an attempt to define the minimal region of the ING1b protein that is capable of inducing high levels of apoptosis in target cells. Adenovirus-mediated expression of ING1b-derived fragments resulted in high cell-killing efficacy in a number of tested cancer cell lines. The identified fragment induced apoptosis in a p53-independent manner, and it displayed strong synergism when used in combination with common HDAC inhibitors. Our findings provide support for using ING1b-derived therapeutics as adjuvant treatments in combination with existing therapies.

## Results

### Overexpression of the truncated version of ING1b protein can induce apoptosis in targeted cells

**Figure 1:**
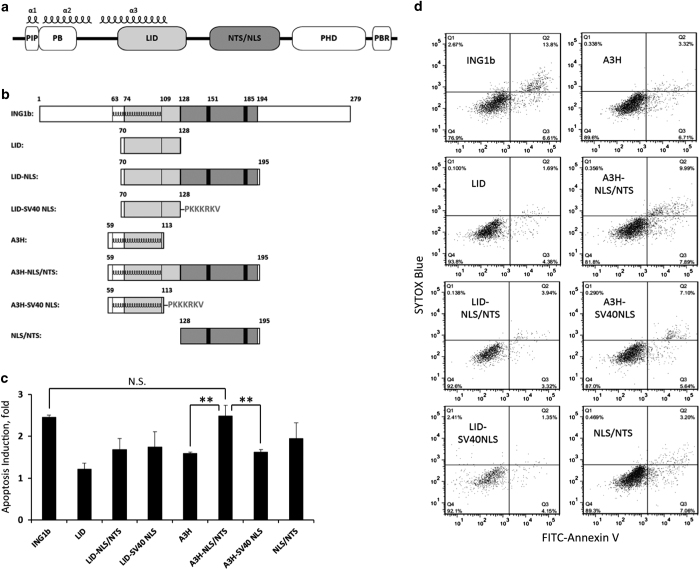
Transient overexpression of ING1b protein fragments induces apoptosis. (**a**) Domain structure of the ING1b protein, where PIP is a PCNA-interacting protein motif, PB the partial bromodomain, LID, a lamin-interacting domain, NLS/NTS, a domain containing two nuclear localization signals and two nucleolar-targeting sequences, PHD, a plant homeodomain, and PBR, the polybasic region that also includes a ubiquitin-interacting motif. Three alpha helixes (*α*1, *α*2, and *α*3) that are highly conserved among all ING family members are indicated that were recently defined by crystallography. (**b**) Diagram showing ING1b fragments used in overexpression experiments. A defined SV40 NTS was also used to target the LID and *α*3 helix (A3H) fragments to the nucleus. Numbers indicate the position of analyzed fragments with respect to full-length ING1b protein; the start positions of the NTS regions are at 151 and 185aa, respectively. (**c**) Induction of apoptosis in HEK-293 cells transfected with DNA vectors encoding the indicated ING1b fragments. An mCherry tracer was used to correct for variable transfection efficiency. Cells were stained 48 h after transfection with FITC-Annexin V and SYTOX blue dyes and analysed by flow cytometry. Data were normalized to the level of apoptosis in cells transfected with the vector containing only the mCherry tracer. Values represent mean±S.D., *n*=3 and ** indicates a significant difference between two means using the Student’s *t*-test with *α*=0.05. NS indicates no significant difference. (**d**) Representative scatter plots from the Annexin V experiments described in **c** that show changes in the size of apoptotic cell populations upon treatment with the indicated ING1b fragments.

To identify the minimal region of ING1b that is still capable of inducing apoptosis, we focused on three structural parts of ING1b protein, the lamin-interacting domain (LID), nuclear localization signal (NLS)/nucleolar-targeting sequence (NTS) domain and *α*3 protein helix (A3H) (Figure 1a). The LID motif has a critical role in maintaining ING1b levels and biological functions in the nucleus.^24^ The NLS/NTS domain mediates subcellular targeting of ING1b and contains two NLSs and two NTSs, respectively.^25^ The NLS/NTS region was recently shown to mediate protein–protein interactions required for ultraviolet-induced apoptosis.^12^ The NLS region of the ING4 protein that is a close homologue of ING1b, physically associates with p53, and these interactions are essential for ING4-induced apoptosis.^20^ Direct protein–protein interactions between ING1b and p53 were also reported; however, the region of ING1b that mediates interaction was not identified.^26^ The A3H region is highly conserved among all ING family members. It largely overlaps with the LID motif and was found to be indispensable for ING4-induced apoptosis.^27^

To test whether ectopic expression of truncated versions of ING1b could induce apoptosis, we designed and cloned expression constructs containing A3H, LID and NLS/NTS motifs (Figure 1b). To facilitate isolation of transfected cells, an mCherry tracer was introduced into the expression vector downstream of the bicistronic element (Supplementary Figure S1). Using Annexin V staining in combination with flow cytometry, we identified an ING1b fragment containing both the A3H and NLS/NTS motifs that induced apoptosis as efficiently as the full-length ING1b protein (Figures 1c and d). Substituting the well-defined SV40 NLS sequence for the ING1-derived NLS/NTS did not show levels of apoptosis comparable to the A3H-NLS/NTS, indicating that the NLS/NTS had functions beyond targeting the construct to the nucleus or nucleolus. This may involve direct association of the region with nucleolar protein CSIG.^12^ These data show that the A3H-NLS/NTS induced apoptosis as effectively as full-length ING1b protein, and that both the A3H and NLS/NTS motifs were required to achieve the full apoptotic effect. They also show that the DNA damage-responsive PCNA-interacting protein (PIP) motif, the partial bromodomain, plant homeodomain (PHD) or polybasic region are not needed for induction of apoptosis.

### Proper localization of ING1b requires a complete NLS/NTS domain

**Figure 2:**
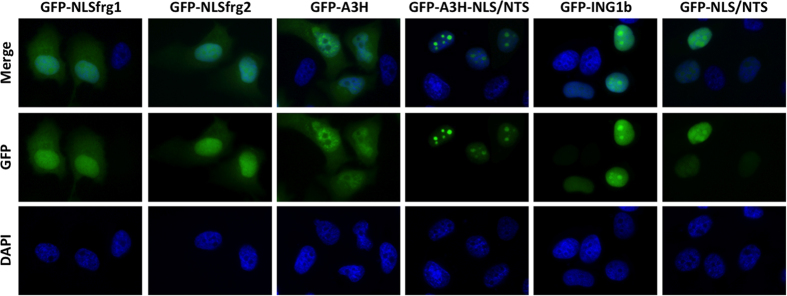
Subcellular localization of ING1b fragments in HeLa cells. Fluorescent images showing subcellular localisation of GFP-fused ING1b fragments in HeLa cells fixed 24 h after transfection. The NLSfrg1 and NLSfrg2 constructs encompass the first and second half of the NLS/NTS domain, with each containing single NLS and NTS motifs, respectively. Magnification is ×630.

Proper subcellular localization of ING1b is essential for its biological functions and the translocation of ING1b to the nucleolus appears to be required for apoptosis.^12,25^ The NLS/NTS domain of ING1b contains two NLS regions, each containing a functional NTS motif.^25^ To better understand the role that these motifs have in ING1b localization, we generated green fluorescent protein (GFP) fusions of full-length ING1b protein and selected ING1b fragments. These included GFP fusions of two halves of the NLS/NTS domain, each containing a single NLS and NTS motif. As anticipated, ING1b and the A3H-NLS/NTS and NLS/NTS fragments showed similar localization pattern and were observed in both the nucleus and nucleolus (Figure 2). One notable difference, however, was that the A3H-NLS/NTS fragment exhibited a significantly stronger bias for localizing to the nucleolus, and it was almost exclusively found there. The localization of the NLS/NTS fragment resembled that of the full-length ING1b protein and produced a strong signal in both the nucleus and nucleolus. The A3H fragment was present throughout the entire cell, but was absent from the nucleolus. As nucleolar targeting may be important for apoptosis, we also fused the A3H fragment with the short NTS sequence derived from the NIK protein.^28^ We observed no substantial difference in cell death between cells transfected with A3H fragment and the A3H-NIK NTS fusion (Supplementary Figure S2). The latter suggests that the low levels of cell death previously observed in cells overexpressing the A3H fragment were not due to A3H fragment exclusion from the nucleolus. The fragments containing either of two halves of the NLS/NTS domain were observed throughout the nucleus as well as in the cytoplasm, but not in the nucleolus. To further corroborate our observations, we generated an ING1b deletion mutant lacking the A3H-NLS/NTS region and examined its localization. As predicted, it was excluded from the nucleolus (Supplementary Figure S3). These data show that ING1b requires a complete NLS/NTS domain containing two NLS and two NTS motifs for efficient loclization to the nucleus and nucleolus.

### Cell death induced by the A3H-NLS/NTS fragment is p53 independent

**Figure 3:**
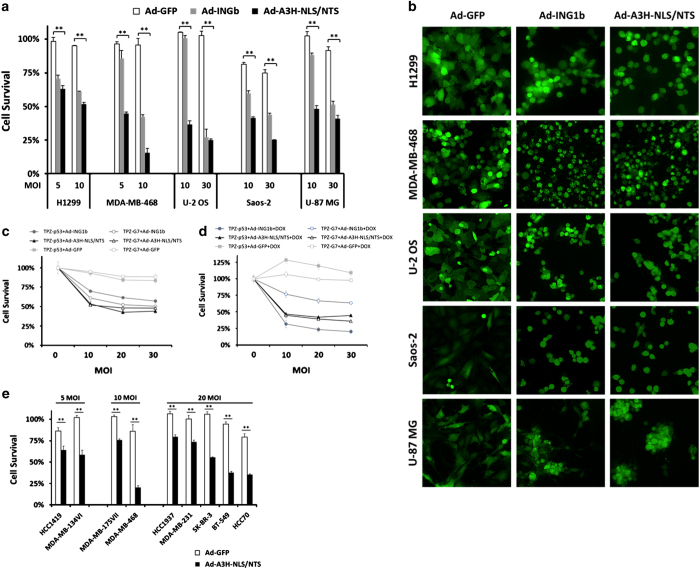
The A3H-NLS/NTS fragment induces cell death in a p53-independent manner. (**a**) H1299, MDA-MB-468, U-2 OS, Saos-2 and U-87 MG cancer cell lines were infected with adenoviruses expressing full-length ING1b or the A3H-NLS/NTS fragment together with a GFP tracer. Cells infected with adenovirus expressing only the GFP tracer were used as a control. Levels of cell death were estimated 48 h after transfection using the MTT assay, and cell survival was normalized to untreated control samples. Values represent mean±S.D., *n*=3 and ** indicates a significant difference between two means using the Student’s *t*-test with *α*=0.05. (**b**) Representative images of cancer cell lines infected with the indicated adenoviral constructs. Cells were visualized using the GFP tracer 48 h after infection. Magnification is ×100. (**c** and **d**) Two H1299-derived isogenic p53-null cell lines containing in their genome either empty vector (H1299 TPZ-G7) or the *p53* gene under the control of a doxycycline-inducible promoter (H1299 TPZ-p53) were grown in media without (**c**) and with (**d**) doxycycline (0.5 *μ*g/ml) supplementation for 36 h before being infected with Ad-GFP, Ad-ING1b or Ad-A3H-NLS/NTS at the indicated MOI. Levels of induced cell death in TPZ-G7 and TPZ-p53 cells were assessed 48 h after infection using an MTT assay. Cell survival was normalized to the untreated control (zero MOI) samples. Values represent mean±S.D., *n*=3. (**e**) Breast cancer cell lines were infected with Ad-GFP or Ad-A3H-NLS/NTS at the indicated MOI. The MTT assay was used to estimate the levels of cell death induced 48 h after transfection. Cell survival was normalized to the untreated control samples. Values represent mean±S.D., *n*=3 and ** indicates a significant difference between two means as estimated by the Student’s *t*-test, *α*=0.05.

The killing efficiency of adenoviral CMV-driven A3H-NLS/NTS fragment was compared directly with CMV-driven Ad-ING1b, both of which expressed GFP under a separate promoter. Adenovirus expressing only GFP protein (Ad-GFP) was used as a control. Five cancer cell lines including non-small cell lung carcinoma (H1299), osteosarcoma (U-2 OS and Saos-2), breast cancer (MDA-MB-468) and glioblastoma (U-87 MG) were used to evaluate the constructs and all lines were sensitive to the full-length and truncated forms of ING1. However, when used at lower multiplicity of infection (MOI) ranges, Ad-A3H-NLS/NTS displayed higher cell-killing compared with Ad-ING1b (Figures 3a and b). Infection at higher MOI diminished this difference and resulted in similar efficacy between the two adenoviruses. ING1b has been reported to induce cell death by both p53-dependent and p53-independent mechanisms. To ask whether Ad-A3H-NLS/NTS-induced cell death was p53 dependent, we used isogenic cell lines derived from the p53-negative H1299 line.^29^ H1299 TPZ-p53 contains a doxycycline-inducible promoter driving p53 expression, whereas H1299 TPZ-G7 contains an empty expression cassette. Cell lines were grown for 36 h either with or without doxycycline, and then infected at different MOIs. Cell survival was estimated by the MTT (3-(4,5-dimethylthiazol-2-yl)-2,5-diphenyltetrazolium bromide) assay 48 h after infection. When grown in the absence or the presence of doxycycline, cell lines showed comparable levels of induced cell death (Figures 3c and d), consistent with the Ad-A3H-NLS/NTS inducing apoptosis in a p53-independent manner. Ad-A3H-NLS/NTS acting independently of p53 is also supported by U-87 MG (p53WT), U-2 OS (p53WT), MDA-MB-468 (p53 mutant), H1299 (p53 mutant) and Saos-2 (p53 null) cells all being sensitive to Ad-A3H-NLS/NTS-induced cell death, and MDA-MB-468 being most sensitive (Figure 3a). In contrast to the A3H-NLS/NTS, full-length ING1 showed an additive effect with p53 on cell killing in some cell lines (Figure 3d). This is consistent with previous studies reporting either additive or synergistic intertactions between p53 and ING1.^23,26,30,31^ It may result from the ability of ING1b to stabilize and modulate the activity of p53 and its target genes.^32–34^ Additionally, nine breast cancer cell lines that differed in p53 status and that showed different sensitivity to HDAC inhibitors (Supplementary Table S1 and data not shown) showed no obvious correlation between the levels of Ad-A3H-NLS/NTS-induced cell death, p53 status and sensitivity to HDAC inhibitors (data not shown). In summary, Ad-A3H-NLS/NTS-induced cell death is p53 independent and is more efficient than Ad-ING1b when used at low MOI.

### Adenoviral delivery of the A3H-NLS/NTS fragment elicits a strong apoptotic response

**Figure 4:**
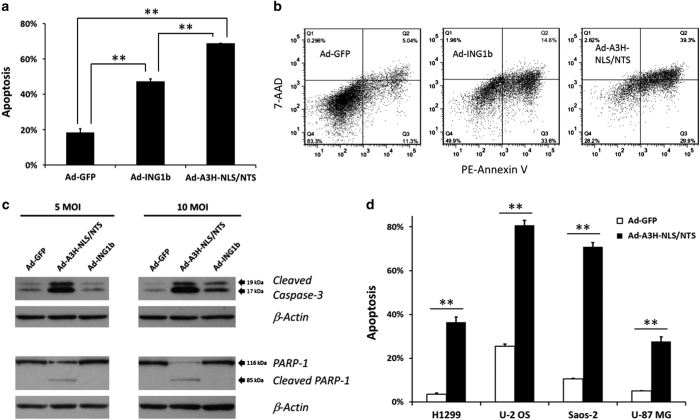
Expression of the A3H-NLS/NTS fragment triggers apoptosis. (**a**) The percentage of apoptotic cells found in populations of MDA-MB-468 breast cancer cells infected at an MOI of 15 with Ad-GFP, Ad-ING1b or Ad-A3H-NLS adenoviruses carrying a GFP tracer. At 48 h after infection, cells were stained with PE-Annexin V and 7-AAD dyes, and levels of apoptosis in GFP-positive cells were measured using flow cytometry. Values represent mean±S.D., *n*=3 and ** indicates a significant difference between two means using the Student’s *t*-test with *α*=0.05. (**b**) Representative scatter plots from the Annexin V experiments described in **a**. (**c**) Immunoblots for caspase-3 and PARP-1 (cleaved and uncleaved) fragments in MDA-MB-468 cells 48 h after infection at 5 and 10 MOI of Ad-GFP, Ad-A3H-NLS/NLS or Ad-ING1b. Membranes blotted for caspase-3 and PARP-1 were stripped and re-blotted with *β*-actin antibodies as a loading control. (**d**) The percentage of apoptotic cells found in H1299, U-2 OS, Saos-2 and U-87 MG cancer cell lines infected at 10 MOI (H1299) or 20 MOI (U-2 OS, Saos-2 and U-87 MG) of Ad-GFP and Ad-A3H-NLS/NTS adenoviruses. Cells were stained with PE-Annexin V and 7-AAD dyes 48 h after infection, and levels of apoptosis in GFP-positive cells were measured using flow cytometry. Values represent mean±S.D., *n*=2 and ** indicates a significant difference between two means as estimated by Student’s *t*-test with *α*=0.05.

Multiple studies have reported that ING1b overexpression induces apoptosis.^19,21,23,35^ As the A3H-NLS/NTS fragment induced cell death, we also measured levels of apoptosis using Annexin V staining and flow cytometry. MDA-MB-468 breast cancer cells infected with Ad-A3H-NLS/NTS at 15 MOI triggered a strong apoptotic response with ~70% of cells undergoing apoptosis 48 h after infection (Figure 4a). Ad-A3H-NLS/NTS was more efficient at inducing apoptosis than Ad-ING1b when measured by Annexin V (Figures 4a and b). Furthermore, cells infected with Ad-A3H-NLS/NTS adenovirus showed significantly increased expression of late apoptotic markers such as cleaved caspase-3 and cleaved PARP-1 fragments (Figure 4c). The induction of apoptosis by Ad-A3H-NLS/NTS and Ad-ING1b was time- and dose dependent, with apoptosis observed 24 h after infection (Supplementary Figure S4). Increasing MOI increased the proportion of cells undergoing apoptosis, but also resulted in elevated non-specific toxicity as shown by increased apoptosis in Ad-GFP-infected control cells. The ability of Ad-A3H-NLS/NTS to elicit a specific apoptotic response at low MOI suggests that it may be used under conditions that minimize off-target effects and reduce non-specific toxicity associated with the use of adenovirus as a delivery vehicle. We also tested Ad-A3H-NLS/NTS-induced apoptosis using H1299, U-2 OS, Saos-2 and U-87 MG cancer cell lines. We found that when infected with Ad-A3H-NLS/NTS, H1299 and U-87-MG cells displayed 5- to 10-fold higher rates of apoptosis when compared with cells infected with Ad-GFP (Figure 4d; Supplementary Figure S5). The high levels of Ad-A3H-NLS/NTS-induced apoptosis in U-2 OS (p53WT) and Saos-2 (p53-null) cancer cell lines confirm its p53-independent nature (Figure 4d; Supplementary Figure S6).

### The Ad-A3H-NLS/NTS and HDAC inhibitors act synergistically to kill cells

**Figure 5:**
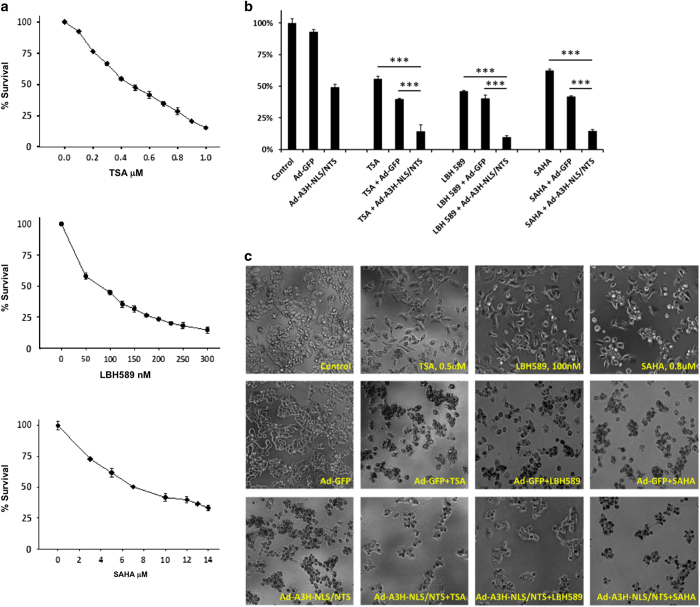
Combining the Ad-A3H-NLS/NTS with HDAC inhibitors. (**a**) Dose–response curves for MDA-MB-468 breast cancer cells treated with TSA, LBH 589 and SAHA. Cells were treated with the indicated HDAC inhibitor for 72 h and the levels of cell death were measured using an MTT assay. Cell survival data were normalized to untreated control samples. Values represent mean±S.D. with *n*=3. (**b**) MDA-MB-468 breast cancer cells were treated with TSA (0.5 *μ*M), LBH 589 (100 nM) or SAHA (0.8 *μ*M) alone, or in combination with Ad-GFP or Ad-A3H-NLS/NTS virus at an MOI of 5. Cells grown without virus or HDAC inhibitors served as controls. For combination treatments, cells were first exposed to drug, and 24 h later infected with virus and incubated an additional 48 h in growth medium supplemented with fresh HDAC inhibitor. Cell survival was assessed using the MTT assay. Values represent mean±S.D. with *n*=3 and *** indicating a significant difference between two means by the Student’s *t*-test with *α*=0.01. (**c**) Representative phase-contrast images of MDA-MB-468 breast cancer cells from **b**. Magnification is ×100.

**Figure 6:**
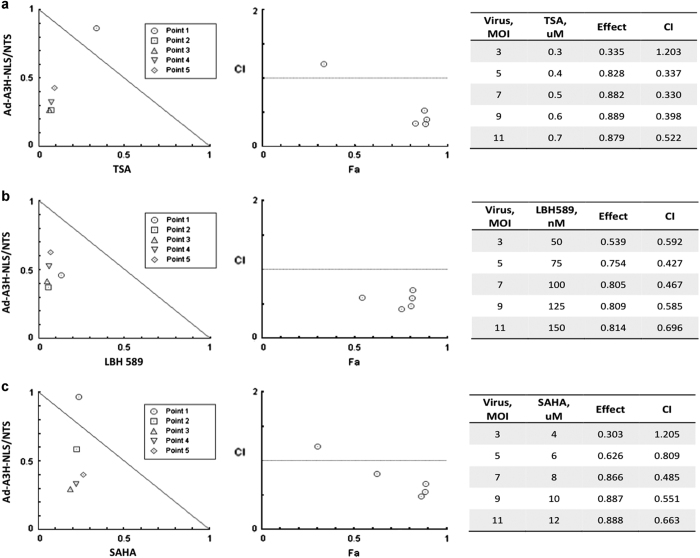
Synergism between Ad-A3H-NLS/NTS and HDAC inhibitors. The strength of synergistic responses when using Ad-A3H-NLS/NTS in combination with either TSA (**a**), LBH 589 (**b**) or SAHA (**c**) HDAC inhibitors was evaluated using CompuSyn software. Five different concentration combinations were selected for each virus–drug pair tested. For each virus–drug pair, an isobologram analysis was performed, and the Fa–CI (Fraction affected—Combination Index) plot was generated. CI values of 0.1–0.3, 0.3–0.7 and 0.7–0.85 indicate strong, medium and modest synergism, respectively.

HDAC inhibitors block deacetylation of histone and other proteins leading to global changes in chromatin structure and transcription, which often culminates in apoptosis. HDAC inhibitors are approved for use in several cancer types and they show significant promise for cancer treatment when combined with other therapeutics. As Ad-A3H-NLS/NTS was able to induce high levels of apoptosis in cancer cells, we tested whether using Ad-A3H-NLS/NTS in combination with HDAC inhibitors would enhance therapeutic efficacy. For combination experiments, we selected vorinostat (SAHA), panobinostat (LBH 589) and trichostatin A (TSA) that are either FDA-approved, in phase III or undergoing preclinical trials. Next, we determined dose–response relations for these HDAC inhibitors in MDA-MB-468 breast cancer (Figure 5a). For combination experiments, MDA-MB-468 cells pre-treated for 24 h with TSA, SAHA or LBH 589 at IC_50_ concentrations, were subsequently infected with Ad-A3H-NLS/NTS at an MOI of 5. Cell survival was estimated by the MTT assay 48 h after infection. Ad-A3H-NLS/NTS significantly increased cell killing by all three HDAC inhibitors (Figures 5b and c). To obtain a more quantitative estimate of synergism between Ad-A3H-NLS/NTS and HDAC inhibitors, we performed drug-combination analysis using normalized isobolograms and combination indices (CIs) calculated with CompuSyn software.^36^ This software is based on the previously developed algorithm for quantization of synergism and antagonism between two drugs.^37^ On the basis of the CompuSyn output, the highest synergism was achieved with Ad-A3H-NLS/NTS and TSA. Among our five tested combinations, two exhibited strong synergism (CI 0.3), and two displayed synergism (CI 0.4–0.5) (Figure 6a). All five tested combinations of LBH 589 with Ad-A3H-NLS/NTS showed synergism with CIs ranging from 0.4 to 0.7 (Figure 6b), and three out of five Ad-A3H-NLS/NTS combinations with SAHA exhibited synergism with CIs of 0.5–0.7 (Figure 6c). These findings emphasize the utility of combined therapies for tumour treatment.

## Discussion

Overexpression studies reported by many groups indicate that restoring ING1b expression in cancer cells to supraphysiological or even physiological levels can trigger cell cycle arrest, inhibit cell growth and induce apoptosis.^19,21,23,35^ In particular, adenovirus-mediated delivery of ING1b and ING4 was used to control growth and dissemination of a variety of cancer cells in mouse xenograft models.^22,38–40^ In these studies, restoring activity of either ING1b, a stoichometric targeting subunit of the mSin3A HDAC complex, or ING4, a stoichometric targeting member of the HBO1 HAT complex,^3^ induced apoptosis, autophagy, triggered cell cycle arrest and inhibited proliferation, angiogenesis and invasiveness of cancer cells. These studies indicate that altering acetylation profiles, either by interfering with acetylation or deacetylation, severely compromises cell viability, particularly in cancer cells.

From our study, it appears that at least one ING-derived peptide is able to affect cancer cell viability as well as the full-length ING1 protein, and in some cases it is actually more effective in killing cells than full-length ING1 via pathways that resemble apoptosis. Ectopic expression of the A3H-NLS/NTS fragment in cancer cells using adenovirus-mediated delivery resulted in a drastic reduction in cancer cell survival, and concommitant increases in apoptotic markers such as Annexin V binding, and PARP and caspase-3 cleavage. Despite being very effective in inducing apoptosis, the mechanism by which the the A3H-NLS/NTS fragment promotes apoptosis is unclear. It does not require the domains or motifs that have been determined to bind PCNA (PIP^25^), 14-3-3 (Ser 199^41^), phosphatidylinositides,^42,43^ ubiquitin^34^ or the H3K4Me3 histone mark.^44^ Although it is somewhat surprising that absence of the PHD did not affect apoptotic activity in our study since it has been previously reported to contribute to the ability of ING1 to induce apoptosis in HT1080 cells,^4^ we would predict that it would be capable of disrupting acetylation since it would block proper targeting of sin3A HDAC complexes to the H3K4Me3 histone mark.^24^

The A3H-NLS/NTS fragment also contains a region previously described as LID that helps retain ING1 in the nucleus through binding lamin A.^24^ If the LID was overexpressed in the nucleus, one would predict that sin3A HDAC complexes would be mislocalized, which is consistent with the highly nucleolar localization that we observe for the A3H-NLS/NTS fragment that contains the LID at its amino terminus. An additional mechanism by which the A3H-NLS/NTS may induce apoptosis is through direct interaction between the nucleolar protein CSIG and ING1 via the NLS/NTS domain that was seen to be necessary for inducing p53-independent apoptosis via the intrinsic apoptosis pathway.^12^ In another study, ectopic expression of an ING1 deletion mutant lacking the PHD but still containing the A3H-NLS/NTS portion of the protein sensitized cells to the extrinsic apoptosis pathway via upregulation of the heat shock protein HSP70.^11^ The upregulation of HSP70 was speculated to disrupt NF-*κ*B survival signalling and trigger TNF-*α*-mediated apoptosis, perhaps through the formation of aggresomes by ING1 proteins. This hypothesis was consistent with their observations, indicating that the N-terminal part of ING1b including a portion of A3H region was prone to form the aggregate due to its hydrophobic nature. Consistent with this idea, a recent ING4 crystallographic study identified the A3H region as one that shows very high structural conservation between ING4 and ING1 and that is necessary for ING4 dimer formation.^27^ If ING1 is indeed capable of forming dimers through regions contained in the A3H-NLS/NTS, expression of this peptide would be expected to act in a dominant-negative fashion and block correct targeting of the sin3A complex, and of other HAT and HDAC complexes if cross-dimerization occurs between ING family members.

Our study has also shown that the A3H-NLS/NTS peptide acts synergistically with three different HDAC inhibitors in inducing cell death in cancer cells (Figure 6). This was not due to an effect of viral oncolysis, as infection with control virus at the same MOI did not induce a significant degree of cell death. Despite this interaction between the peptide and HDAC inhibitors that target the pathways by which ING proteins exert a major part of their cellular effects, combining the A3H-NLS/NTS peptide with other agents directed towards the epigenome such as DNA methyltransferase inhibitors^22^ or other agents such as doxorubicin or radiation may produce even stronger synergistic effects to promote apoptosis of cancer cells.^18,21–23^ Given that such synergies exist between the ING1 fragment and diverse cancer therapeutics, there appears to be a plausible argument for using Ad-A3H-NLS/NTS, as it should improve the therapeutic value of existing therapies, and might also help to reduce treatment side effects if it is possible to maintain treatment efficacy while lowering the doses of therapeutic compounds used. Further studies using mouse preclinical models will help to confirm whether this idea may have clinical value.

## Materials and Methods

### Cell culture

All human cancer cell lines used in experiments were purchased from the ATCC (Manassas, VA, USA). BT-549, H1299, HCC 1419, HCC 1937, HCC 70, HeLa, MDA-MB-134 VI, MDA-MB-175 VII, MDA-MB-231 and MDA-MB-468 breast cancer cell lines were grown in RPMI-1640 medium. SK-BR-3, U-2 OS and Saos-2 cell lines were grown in McCoy's 5a medium (modified). U-87 MG cells were grown in Eagle's minimum essential medium. All growth media were supplemented with 10% FBS, growth factors and antibiotics as per ATCC recommendations. Cells were maintained in a humidified atmosphere at 37 °C and 5% CO_2_ and routinely tested negative for mycoplasma. Growth media were changed every 2–3 days.

### Generation of expression constructs and cell transfections

All expression constructs for ING1b fragment analysis were generated using pcDNA3.1+ vector (Invitrogen, Waltham, MA, USA). The vector was modified by replacing the original MCS with an IRES:mCherry cassette. A new MCS was introduced upstream of the expression cassette and used for cloning ING1b fragments. Introducing the IRES element allowed bicistronic expression of ING1b fragments and mCherry tracer in the same cell, permitting efficient isolation of transfected cells by flow cytometry. For localization experiments, GFP-fused and FLAG-tagged ING1b fragments were cloned into pcDNA3.1+ vector (Invitrogen). For transfections, cells were seeded in six-well tissue culture plates 16 h before transfection. The next day, HEK-293 cells at 80% confluence were transfected using TransIT-293 Transfection Reagent (Mirus, Madison, WI, USA) according to the manufacturer's protocol. HeLa cells were transfected using Lipofectamine 2000 reagent (Invitrogen) as per the manufacturer's protocol. Depending on the experiment, expression levels of transfected constructs were analyzed 24 and/or 48 h after transfection.

### *In vivo* localization experiments

GFP-fused ING1b fragments were transfected into HeLa cells plated on coverslips. At 24 h after transfection, cells were fixed with 4% paraformaldehyde in phosphate-buffered saline (PBS), permeabilized with 0.5% Triton X-100, and stained with 4′,6-diamidino-2-phenylindole (DAPI). FLAG-tagged ING1b fragments were transfected into HEK-293 cells grown on coverslips. At 48 h after transfection, cells were fixed with 4% paraformaldehyde in PBS and permeabilized with 0.5% Triton X-100. The FLAG-tag was visualized by immunofluorescence using mouse monoclonal anti-FLAG primary antibodies (Sigma, St. Louis, MO, USA) followed by goat anti-mouse Alexa 488-conjugated secondary antibodies (Invitrogen). The nucleolar protein fibrillarin was visualized using rabbit polyclonal anti-fibrillarin primary antibodies (Santa Cruz, Dallas, TX, USA), followed by donkey anti-rabbit Alexa 568-conjugated secondary antibodies (Invitrogen). DNA was stained with DAPI (1μg/ml). After staining, coverslips were mounted on glass slides and examined under the Axio inverted microscope with AxioVision v4.8 software (Zeiss, Oberkochen, Germany).

### Generation of adenovirus constructs

The adenoviral construct for expression of A3H-NLS/NTS fragment was generated using the pAd-Easy system^45^ according to the published protocol.^46^ Generation of ING1b- and GFP-expressing adenoviruses was previously described.^22^ For A3H-NLS/NTS-expressing adenovirus, the A3H-NLS/NTS fragment was cloned into the pAdTrack-CMV vector (Invitrogen) containing a GFP tracer under the control of the CMV promoter. The resulting vector was recombined with pAdEasy-1 in BJ5183-AD-1 electroporation-competent cells (Stratagene, Santa Clara, CA, USA). Recombinant clones were verified by enzymatic digestions. Next, purified recombinant adenovirus plasmid was linearized with PacI (NEB, Ipswich, MA, USA) and introduced into the packaging cell line, HEK-293, by lipofection using TransIT-293 Transfection Reagent (Mirus). Following the series of virus amplification steps carried out in HEK-293 cells, adenoviruses were purified, and the virus titre was measured using a fluorescent focus assay.

### Apoptosis assays

Levels of induced apoptosis were measured using the Annexin V assay followed by flow cytometry. For experiments that involved lipid-mediated DNA delivery, HEK-293 cells were seeded in six-well tissue culture plates 16 h before transfection. The following day, HEK-293 cells at about 80% confluence were transfected using TransIT-293 Transfection Reagent (Mirus) as per the manufacture’s protocol. At 48 h after transfection, cells were harvested, washed twice with cold PBS and resuspended in 1× Annexin V binding buffer (BioLegend, San Diego, CA, USA) at a concentration of 1×10^6^ cells/ml. Next, cells were stained with FITC, Annexin V (BioLegend) and SYTOX Blue (Invitrogen) dyes according to the manufacturer’s protocols, incubated in the dark at room temperature for 15 min and analysed by flow cytometry within 1 h (Flow Cytometry Facility at the University of Calgary). The mCherry tracer was used to identify transfected cells. Levels of induced apoptosis in cells transfected with INGb fragments were normalized to the level of apoptosis in cells transfected with the vector containing only the mCherry tracer. For adenovirus-induced apoptosis, ~2.5×10^5^ cells were plated per well in six-well tissue culture plates 16 h before infection with either Ad-GFP (control), Ad-ING1b or Ad-A3H-NLS/NTS adenovirus carrying the GFP tracer. Depending on the experiment, cells were collected 24 or 48 h after infection, washed twice with cold PBS and resuspended in 1× Annexin V binding buffer (BioLegend) at a concentration of 1×10^6^ cells/ml. Cells were stained with PE-Annexin V (BioLegend) and 7-AAD (BioLegend) dyes according to the manufacturer’s protocols and incubated in the dark at room temperature for 15 min. The levels of apoptosis in GFP-positive cells were measured using flow cytometry within 1 h (Flow Cytometry Facility at the University of Calgary).

### MTT assays

Cell survival following adenovirus infection was evaluated using the MTT assay.^47^ Approximately 1.5×10^4^ (H1299; TPZ-G7, TPZ-p53, U-2 OS, Saos-2), 2.5×10^4^ (U-87 MG) and 3×10^4^ (BT-549, HCC 1419, HCC 1937, HCC 70, MDA-MB-134 VI, MDA-MB-175 VII, MDA-MB-231, MDA-MB-468, SK-BR-3) cells, respectively, were plated per well in 96-well tissue culture plates 16 h before experiments. All treatments were done in triplicate, and at least three wells per plate were left without cells and served as blanks for absorbance measurements. At 48 h after infection, growth medium was removed and 50 *μ*l of MTT stock in PBS (5 mg/ml) was added to each well, including blank wells. The plates were then incubated in the dark for 4 h at 37 °C. Following incubation, 150 *μ*l of DMSO was added to each well with MTT to solubilize formazan crystals. To ensure complete solubilisation, plates were incubated with gentle agitation for an additional 10 min at 37 °C. Absorbance was measured at 540 nM (formazan) and 720 nM (background) with a Bio-Rad microplate reader (Mississauga, ON, Canada). To correct for background noise, the 720 nM OD background reading was subtracted from 540 nM OD total signal. Cell survival was calculated using the formula (OD treated well−blank_average_)/(OD control well−blank_average_)×100.

### p53 expression experiments

To investigate the role of p53 expression in Ad-A3H-NLS/NTS-induced apoptosis, a doxycycline-regulated inducible p53 expression system consisting of two isogenic H1299-derived cell lines was used.^29^ The TPZ-G7 and TPZ-p53 null cell lines were stably modified with either an empty control vector or p53 gene under the control of the doxycycline-inducible promoter. Although growing these cells in the presence of doxycycline would convert TPZ-p53 into a p53-positive cell line, TPZ-G7 cells remain p53 negative. Without doxycycline treatment both cell lines remain p53 negative.^29^ To induce p53 expression, cells were grown for 36 h in medium supplemented with doxycycline (0.5 *μ*g/ml). Doxycycline-containing medium was changed daily. Following 36 h of induction, cell were collected and seeded in 96-well tissue culture plates for cell survival experiments.

### Western blotting

Cells were collected 48 h after infection with adenovirus, and total cell lysates were prepared using RIPA buffer supplemented with protease inhibitors. Protein concentration in samples was determined using the DC Protein Assay (Bio-Rad) as per the manufacturer's protocol. Equal amounts of total protein from samples were denatured by boiling in Laemmli buffer, resolved using SDS-PAGE and transferred onto nitrocellulose membrane (Millipore, Darmstadt, Germany). The expression of cleaved caspase-3 and PARP-1 (cleaved and uncleaved) fragments was determined using *α*-cleaved caspase-3 (Cell Signaling, Danvers, MA, USA) and *α*-PARP-1 (Santa Cruz) primary antibodies. To quantify loading of samples, all membranes were stripped and re-blotted with anti-*β* actin antibodies (Santa Cruz).

### Treatment with HDAC inhibitors and evaluation of synergism

To generate dose–response curves and to obtain IC_50_ values for MDA-MB-468 breast cancer cells treated with vorinistat (SAHA), panobinostat (LBH 589) and TSA, ~2×10^4^ cells were plated per well in 96-well tissue culture plates 16 h before experiments. The next day, fresh growth medium supplemented with drugs was added. After 24 h of drug treatment, growth media were changed and fresh drugs were added. Following 72 h of drug treatment, cell survival was evaluated using the MTT assay and data were normalized to untreated control samples. For combined virus–drug treatments MDA-MB-468 breast cancer cells were treated with TSA, LBH 589 or SAHA at 0.5 *μ*M, 100 nM and 0.8 *μ*M final concentrations, respectively. Following 24 h of treatment with drugs, cells were infected at an MOI of 5 with Ad-A3H-NLS/NTS or Ad-GFP and grown for an additional 48 h. At the time of infection, fresh growth medium containing drugs was added. Untreated cells, cells only infected with adenovirus and cells only treated with HDAC inhibitors served as controls. Cell survival was assessed using the MTT assay and data were normalized to untreated control cells. To evaluate the strength of synergism between Ad-A3H-NLS/NTS and HDAC inhibitors, we tested five different virus–drug concentration combinations for each HDAC inhibitor, and analysed cell survival data using CompuSyn software (ComboSyn, Inc. Paramus, NJ, USA^36^). Quantitative synergism assessment by CompuSyn was based on normalized isobologram analysis and calculation of the CI. The Cl values of 0.1−0.3, 0.3−0.7 and 0.7−0.85 define strong, medium and modest synergism, respectively. The CI values of 0.9–1.1 show nearly additive effect, and CI values of 1.1–1.2 indicate slight antagonism.

### Statistical analyses

Statistical analyses were performed using JMP 5.0 software (SAS Institute Inc., Cary, NC, USA). In all cases, means and S.D. were calculated. To test for the significance of a difference between two means the two-tailed Student’s *t*-test with *α*=0.05 or *α*=0.01 was used, as indicated.

## Abbreviations

A3H: *α*3 protein helix
CI: combination index
GFP: green fluorescent protein
HAT: histone acetyltransferase
HDAC: histone deacetylase
ING: inhibitor of growth
LID: lamin-interacting domain
MOI: multiplicity of infection
NLS: nuclear localization signal
NTS: nucleolar-targeting sequence
PBS: phosphate-buffered saline
PHD: plant homeodomain
PIP: PCNA-interacting protein
TNF: tumour necrosis factor
TSA: trichostatin A.
