# Supplementary figures and images for: Defining the minimal peptide sequence of the ING1b tumour suppressor capable of efficiently inducing apoptosis
Source: Cell Death Discov. 2015 Oct 26;1:15048–. doi: 10.1038/cddiscovery.2015.48 (PMC4979497; doi:10.1038/cddiscovery.2015.48)

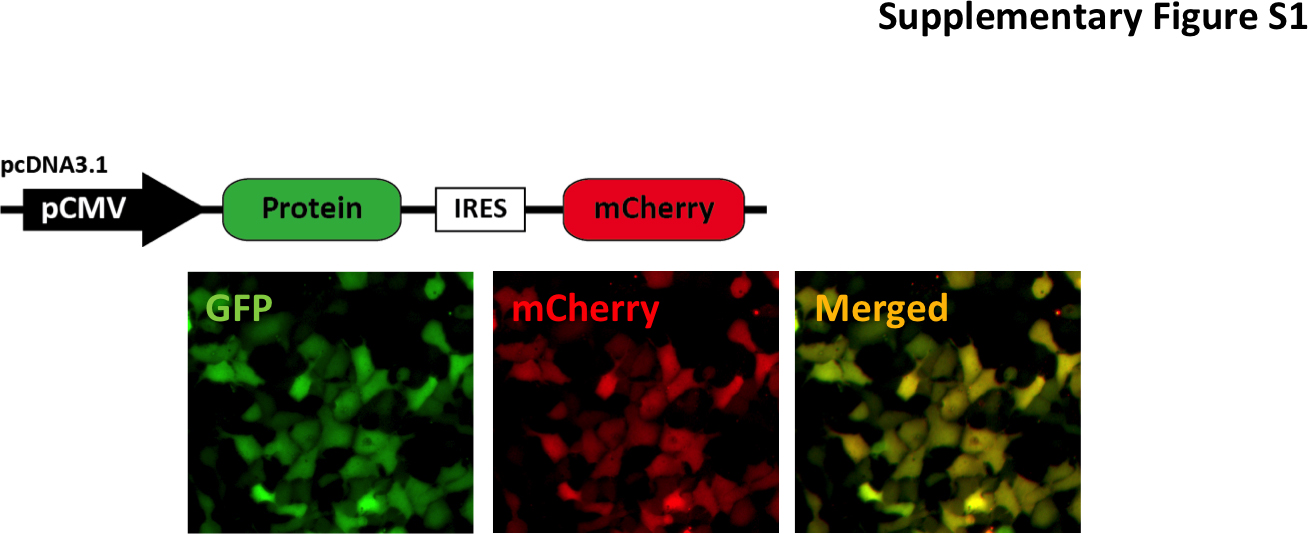

Supplement: Supplementary Figure 1 [file cddiscovery201548-s1.jpg]

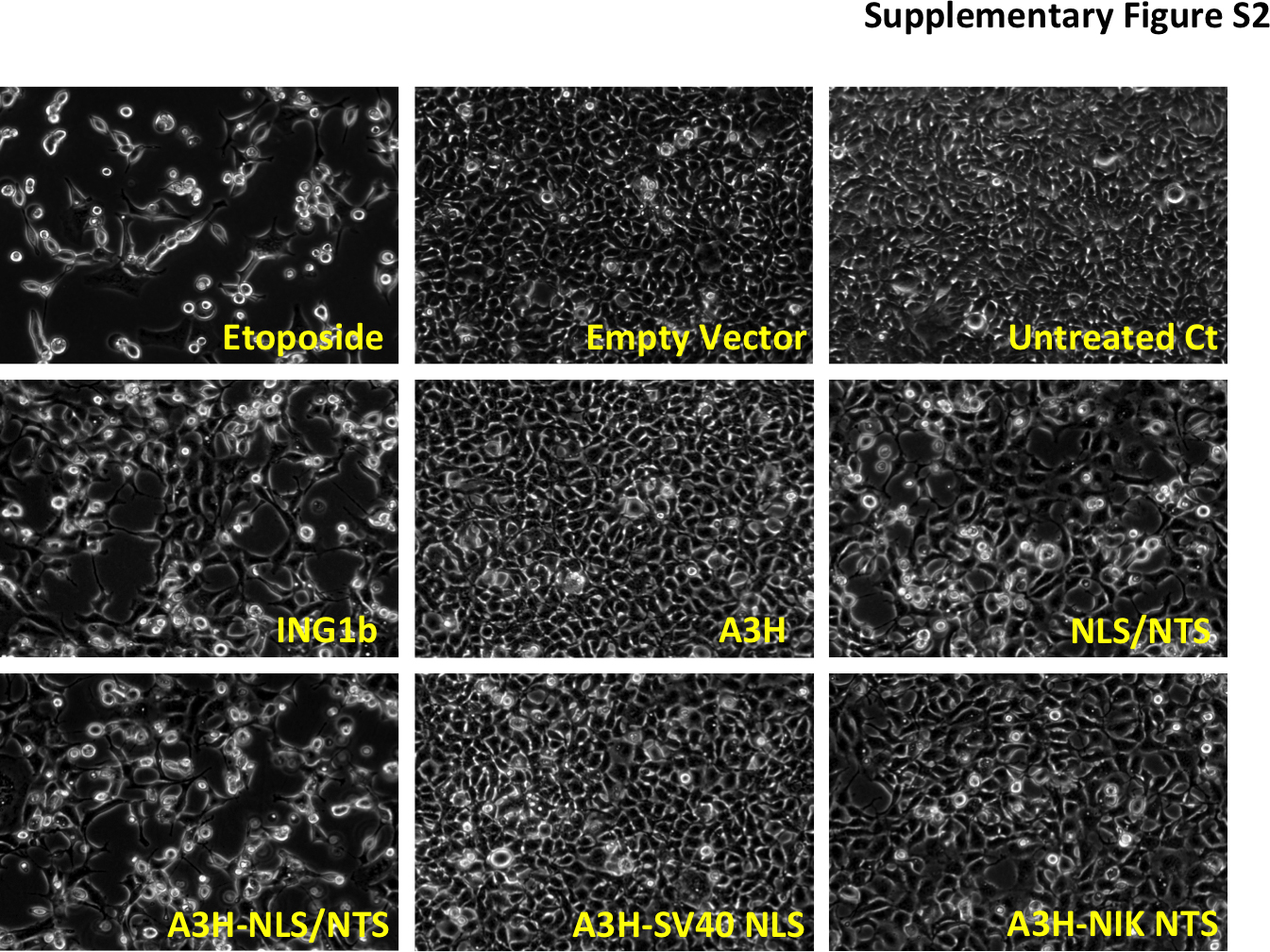

Supplement: Supplementary Figure 2 [file cddiscovery201548-s2.jpg]

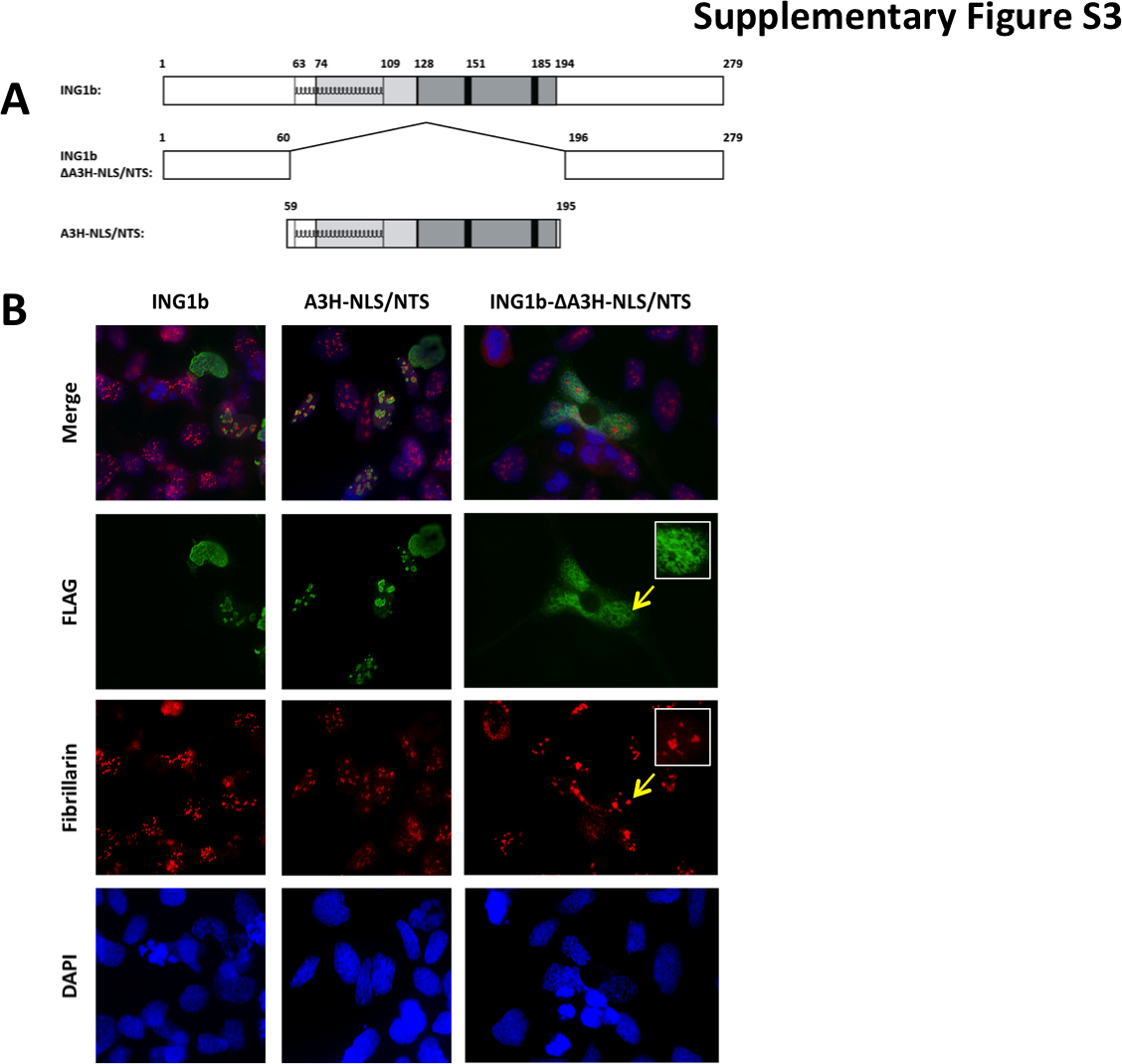

Supplement: Supplementary Figure 3 [file cddiscovery201548-s3.jpg]

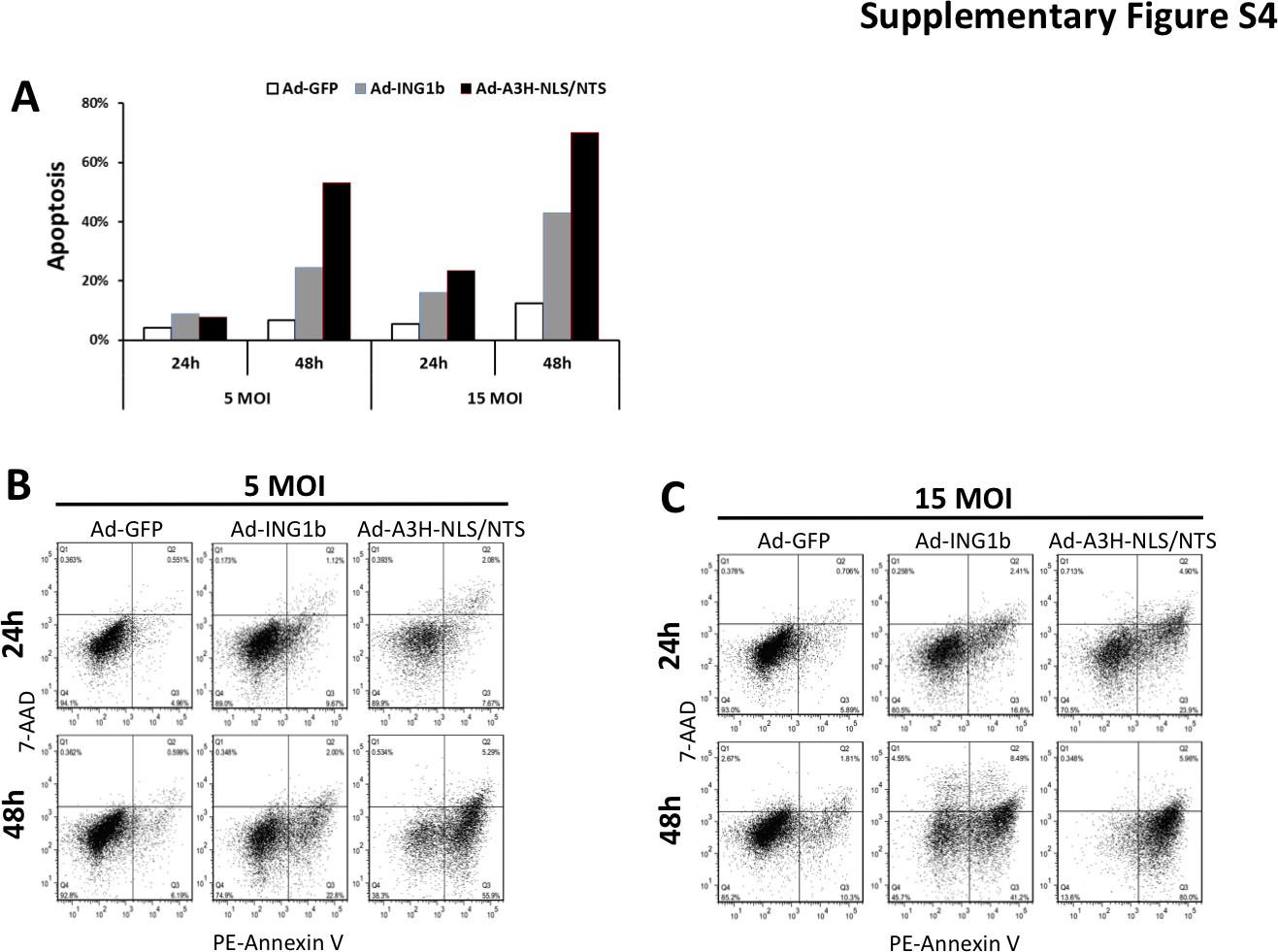

Supplement: Supplementary Figure 4 [file cddiscovery201548-s4.jpg]

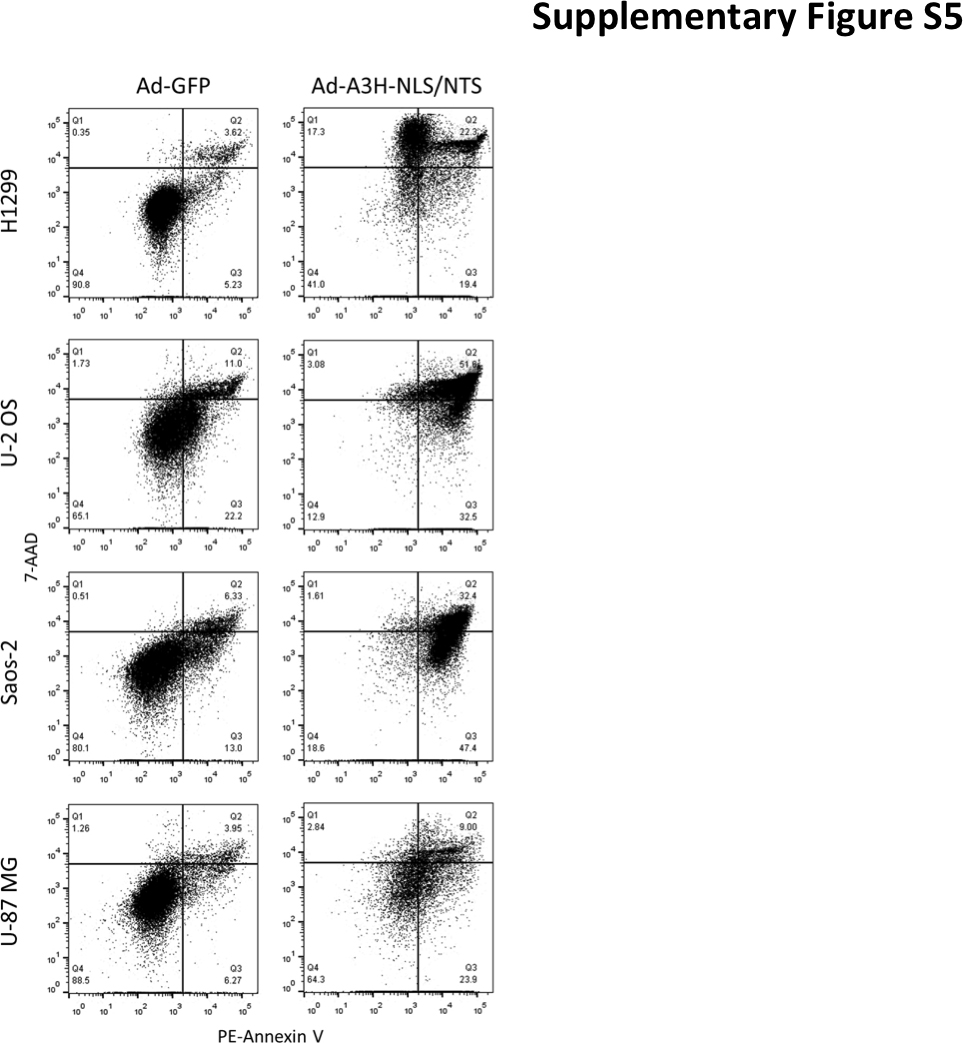

Supplement: Supplementary Figure 5 [file cddiscovery201548-s5.jpg]

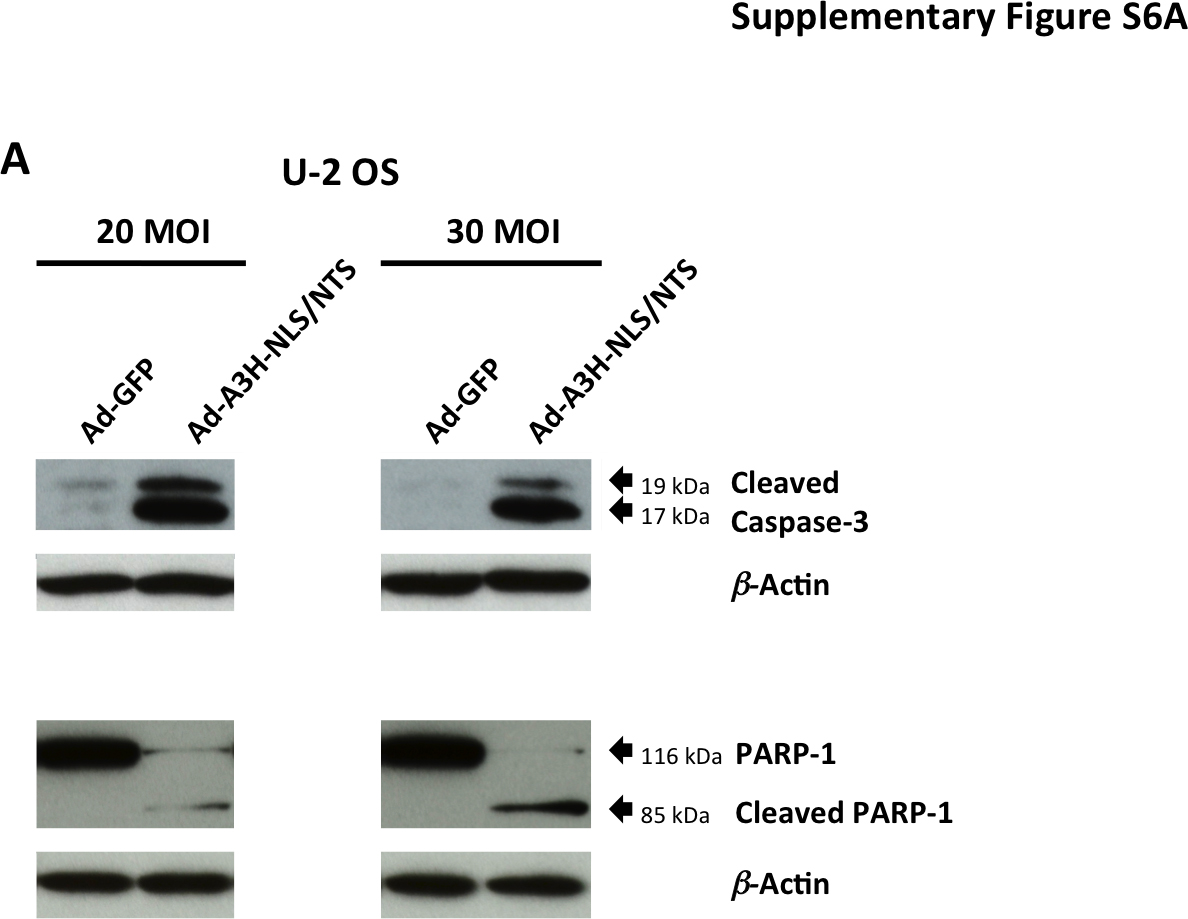

Supplement: Supplementary Figure 6 [file cddiscovery201548-s6.jpg]

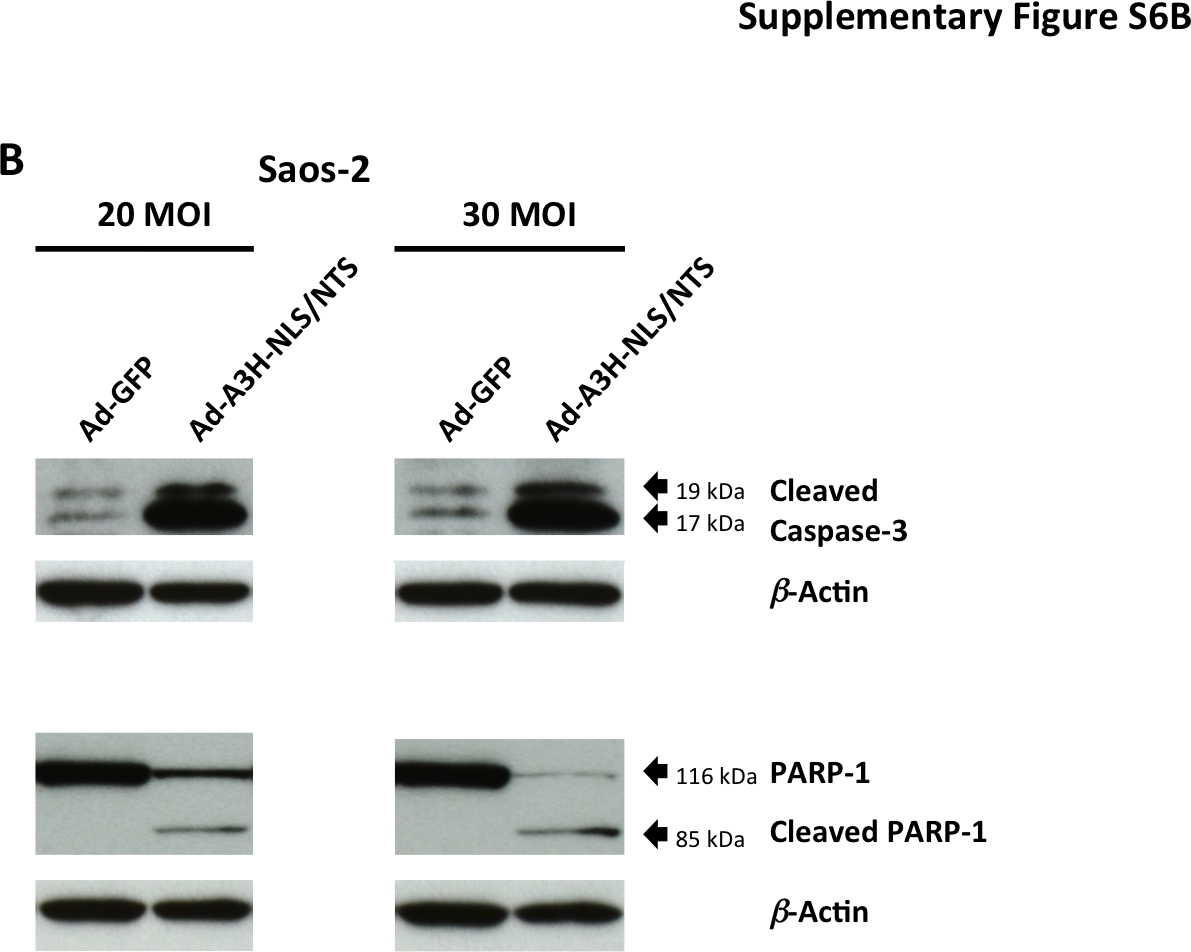

Supplement: Supplementary Figure 7 [file cddiscovery201548-s7.jpg]

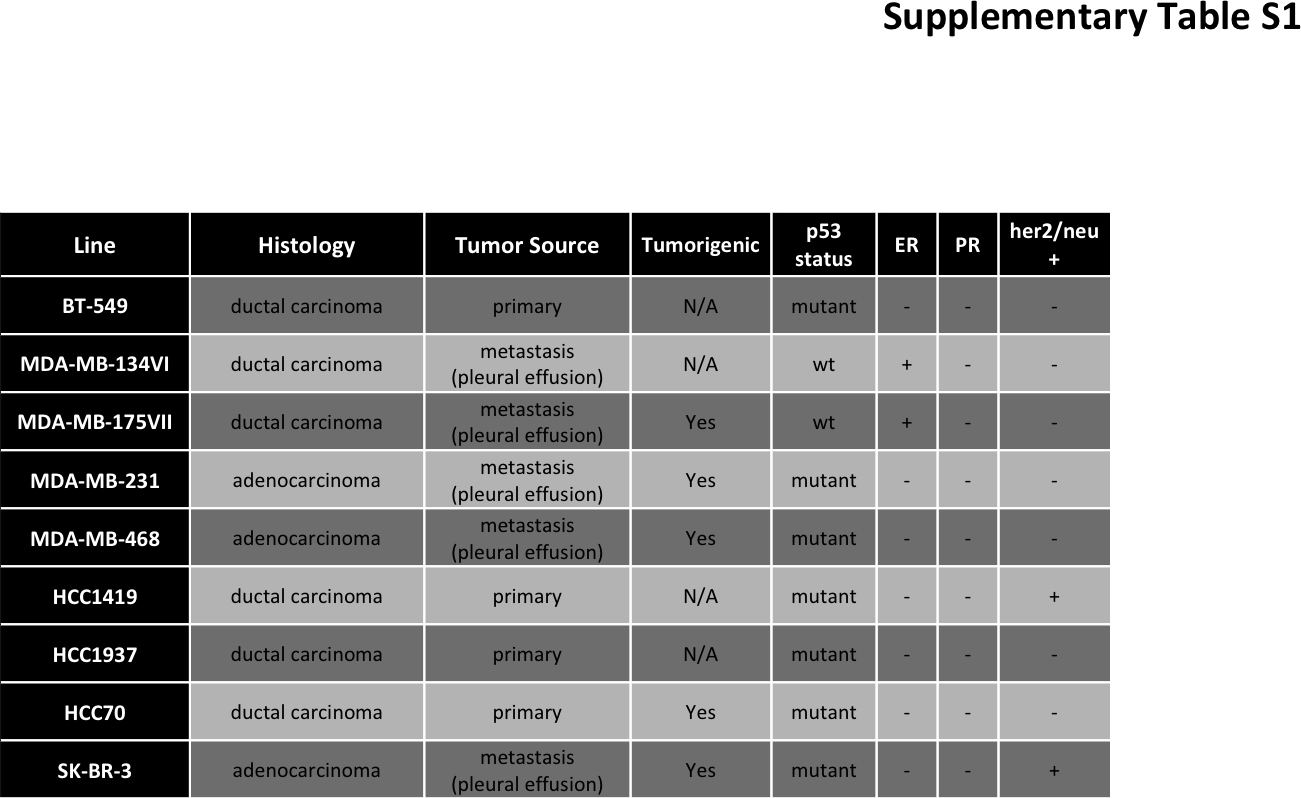

Supplement: Supplementary Table [file cddiscovery201548-s8.jpg]
